# Fall-related efficacy is a useful and independent index to detect fall risk in Japanese community-dwelling older people: a 1-year longitudinal study

**DOI:** 10.1186/s12877-019-1318-5

**Published:** 2019-10-29

**Authors:** Naoto Kamide, Yoshitaka Shiba, Miki Sakamoto, Haruhiko Sato, Akie Kawamura

**Affiliations:** 1 School of Allied Health Sciences, Kitasato University, 1-15-1 Kitasato, Minami-ku, Sagamihara, Kanagawa 252-0373 Japan; 2 Graduate School of Medical Sciences, Kitasato University, Sagamihara, Japan

**Keywords:** Accidental falls, Aged, Fall-related efficacy, Japanese, Physical performance

## Abstract

**Background:**

Fall-related efficacy has been found to be associated with both falls and fall risk factors such as physical performance. The aim of the present study was to clarify whether fall-related efficacy is, independent of physical performance and other potential risk factors, associated with future falls in community-dwelling older people.

**Methods:**

The study participants were 237 Japanese older people aged 65 years and over who were living independently in their community. Fall-related efficacy and physical performance were assessed at baseline using the short version of the Falls Efficacy Scale-International (short FES-I) and 5-m walking time, the Timed Up and Go Test, the 5 Times Sit to Stand Test, and grip strength. Physical performance was then again assessed at 1-year follow-up. The number of falls was obtained every 6 months for 1 year after the baseline survey. Instrumental activities of daily living (IADL), depression, fall history, current medications, medical history, and pain were also investigated as potential confounding factors that have possible associations with falls. The associations between the short FES-I, physical performance, and number of falls were analyzed using Poisson regression analysis adjusted for physical performance and potential confounding factors.

**Results:**

The mean age of the participants (75.9% women) was 71.1 ± 4.6 years, and 92.8% could perform IADL independently. The total numbers of falls and fallers during the 1-year follow-up period were 70 and 42, respectively. On Poisson regression analysis adjusted for walking time and potential confounding factors, independent of physical performance, the short FES-I was found to be significantly associated with number of falls (relative risk = 1.09, *p* < 0.05). On the other hand, physical performance was not significantly associated with the number of falls.

**Conclusions:**

The findings of the present study suggest that the short FES-I, independent of physical performance and other potential risk factors, is a useful index to detect fall risk in community-dwelling older people, and that fall-related efficacy is an important factor in terms of fall prevention.

## Background

Falling is well-known as a representative adverse health outcome that occurs commonly in older people. Indeed, about 20% of Japanese community-dwelling older people have experienced one or more falls over a 1-year period [1, 2]. Further, falling is the main causal factor of serious injuries such as hip fractures [3, 4], and it also causes functional decline [4, 5]. Therefore, prevention of falls is a critical issue for prolonging healthy life expectancy of older people.

In order to prevent falls in older people, it is indispensable that the older individuals who have a high fall risk are effectively identified by fall risk evaluation. In terms of fall risk evaluation, assessment of psychological and physical aspects is important. As for the psychological aspects, fall-related efficacy is a well-known risk factor for falls in older people [6]. Fall-related efficacy can be measured using several assessment scales developed previously; for instance, the Falls Efficacy Scale [7, 8], the Modified FES [9], the Falls Efficacy Scale International (FES-I) [10], and the Activities-Specific Balance Confidence Scale [11] are widely used as representative scales with reliability and validity [6, 12, 13]. These scales have been shown to be associated with a history of falls in cross-sectional studies [10, 14–16]. Furthermore, prospective cohort studies previously indicated that low fall-related efficacy that was discriminated by the FES or FES-I was independently associated with an increased risk of future falls in older people [17–20]. In addition, fall-related efficacy was reported to be not only a fall risk, but also associated with activities of daily living (ADL), social participation, life space, and physical activity [8, 21–24]. Therefore, the assessment of fall-related efficacy in older people is worthwhile to prevent not only falls, but also other negative health outcomes.

On the other hand, fall-related efficacy has also been found to be associated with physical performance, which is well-known to be an important factor for fall risk in older people [12, 21]. In particular, associations between fall-related efficacy and gait and balance function have been reported in previous studies [7, 12, 18, 25–27]. Furthermore, it has been suggested that not only physical performance, but also some other risk factors affect falls [28]. For example, neuropsychological factors (e.g., cognitive function and depression), environmental factors (e.g., lighting of a room, loose carpets, and lack of room safety equipment), ADL, and polypharmacy have been identified as possible risk factors for falls [29]. A previous study indicated that fall-related efficacy interacted with these fall risk factors, in addition to physical performance [18]. That is, physical performance and other potential risk factors may have an effect on the association between fall-related efficacy and fall risk. Thus, to clarify the clinical importance of fall-related efficacy for fall risk, verifying the association between fall-related efficacy and fall risk, taking into account the effects of physical performance and other potential risk factors, is necessary.

The aim of the present study was to clarify whether fall-related efficacy, independent of physical performance and other potential risk factors, is associated with future falls in community-dwelling older people in a longitudinal, observational study.

## Methods

### Participants

The participants of this longitudinal study were recruited from the older people aged 65 years and over who participated in health check-ups for geriatric syndrome organized from 2016 by a university research team and a community sports facility at Sagamihara City, Kanagawa prefecture, in Japan. The recruitment of the participants was performed using advertisements in newspapers and community newsletters. Older people interested in the health check-ups contacted our research center located in the sports facility by mail or telephone, and they were screened for eligibility by the staff of the research center. The health check-ups were held every 6 months, and the baseline data obtained from the 519 older people of the health check-ups held from September, 2016 to September, 2017 were included in this study. However, due to limited personnel and research funding, of the 519 people, the present study finally included only the 237 participants who could participate in a 1-year follow-up survey after the baseline survey. The people who could not be contacted within a definite period or participate in the designated survey schedule were excluded. All participants of this study were aged 65 years and over and were living independently in their community. The eligibility criteria for the present study were: older people who were able to perform ADL independently and who could independently attend the location of the research center located in the sports facility for the present study. The participants’ ADL levels were confirmed by interviews at the health check-up. Individuals who lacked long-term care insurance certification in Japan were considered independent in ADL. Participants suspected of having dementia based on interviews with researchers at a health check-up were excluded.

The present study was approved by the Institutional Review Board of the School of Allied Health Sciences at Kitasato University (approval number 2018-008B), and written, informed consent was obtained from all participants.

### Fall-related efficacy

Fall-related efficacy was assessed using the short version of the FES-I (short FES-I) at the baseline survey [30]. The short FES-I was developed as a shortened version of the FES-I, and it has been translated into many languages including Japanese, and the reliability and validity of the Japanese version have been confirmed [31]. The short FES-I has 7 items, and each item is rated on a four-point Likert scale. Short FES-I scores are obtained by summing the response values (from 1 to 4) for each item. The total score ranges from 7 to 28 points, with lower scores indicating better fall-related efficacy.

### Physical performance

As assessments of physical performance, 5-m walking time, the Timed Up and Go Test (TUGT) [32], the 5 Times Sit to Stand Test (FTSTS) [33], and grip strength were measured at two time points, baseline and at 1-year follow-up. The 5-m walking times were measured under two conditions, at a comfortable pace (5CWT) and at maximum pace (5MWT), using a 9-m walkway including acceleration and deceleration zones that were 2-m each. In addition, the passing time for the 5-m length in the middle of the walkway in each condition was measured as 5CWT and 5MWT, respectively. For measurement of 5CWT, subjects were instructed to walk straight at a “usual” pace. For the measurement of 5MWT, subjects were instructed to walk in a straight line as fast as possible. For the TUGT, the researchers instructed the participants to stand up from a chair without hand support, walk 3 m as quickly as possible, turn around, walk back, and then sit down again [34]. The time required to complete the task was measured as TUGT. FTSTS was conducted in accordance with a previous study [33], using a standard chair (height of 42 cm) without arm rest. The researchers instructed the participants to stand up and sit down with their arms crossed in front of the chest, and to repeat that motion 5 times as quickly as possible. The time required to complete the task was measured as FTSTS. The 5-m walking time, TUGT, and FTSTS were measured using a digital stopwatch (ALBA W072; Seiko Watch Corporation, Tokyo, Japan). Grip strength was measured in the dominant hand using a Smedley-type dynamometer (T.K.K.5401, TAKEI Scientific Instruments Co., Ltd., Niigata, Japan) in the standing position.

### Falls

The number of falls was defined as the primary outcome measure in this longitudinal study. A fall was defined as unintentionally coming to rest on the ground, the floor, or other lower level [35]. The number of falls was obtained using a self-report questionnaire every 6 months at the health check-ups at the research center for 1 year after the baseline survey. Further, regarding previous fall history, the presence or absence of falls during the previous 6 months was also investigated using the self-report questionnaires at baseline. In this study, participants who had fallen two or more times during 1-year follow-up were defined as “recurrent fallers”. Recurrent fallers were studied, because it has been suggested that single fallers are similar to non-fallers in a comparison with recurrent fallers with respect to fall risk factors [36]. This definition and the number of falls were used for further statistical analysis to investigate the associations between falls and the short FES-I scores and physical performance.

### Confounding factors

As potential confounding factors, IADL, depressive symptoms, number of medications, medical history, and pain were investigated. Similarly, height, weight, and body mass index (BMI) were recorded. To assess IADL, a subscale of the Tokyo Metropolitan Institute of Gerontology Index of Competence [37] was used, with scores ranging from 0 to 5 points, and full marks (5 points) indicating independence in IADL. Depressive symptoms were measured using the five-item version of the Geriatric Depression Scale (5-GDS) [38]. Scores on the 5-GDS range from 0 to 5 points, and according to a previous study [38], participants with scores of 2 or more points on the 5-GDS are defined as “with depressive symptoms,” and those with scores of zero or one point are defined as “without depressive symptoms”. With respect to information about number of medications, medical history, pain, presence or absence of knee and low back pain, history of stroke, heart disease, diabetes mellitus, and respiratory disease, and the number of types of drugs taken daily were investigated using self-report questionnaires.

### Statistical analysis

In this study, many subjects could not be included in the follow-up survey due to limitations of personnel and research funding. Therefore, to verify the presence or absence of bias between follow-up subjects and non-follow-up subjects, the differences between the two sets of subjects were statistically analyzed for all variables. The differences between the two groups were analyzed using unpaired *t*-tests for continuous variables such as physical performance tests, the Mann-Whitney U test for ordinal scales such as the short FES-I, and the chi-squared test for categorical variables.

The associations between the number of falls and the short FES-I and physical performance were analyzed using Poisson regression analysis adjusted for age and sex, which were considered strong potential confounding factors. In addition, the associations between number of falls and confounding factors were also investigated using Poisson regression analysis adjusted for age and sex. Finally, in order to determine whether the short FES-I, independent of physical performance, was associated with the number of falls, Poisson regression analysis was performed, adjusting for age, sex, and potential confounding factors, with number of falls set as the dependent variable, and the short FES-I and each physical performance test set as independent variables. The potential confounding factors were derived from the factors associated with the number of falls with a probability of < 10% based on Poisson regression analysis adjusted for age and sex. Model fitting of the Poisson regression analysis in this study was checked using a goodness of fit test [39]. If the probability of the goodness of fit test was greater than 5%, model fitting using Poisson regression was considered acceptable. In addition, the participants were divided into two groups, the recurrent fallers group and the non-recurrent fallers group, according to the definitions above. Receiver operating characteristic (ROC) curves were used to assess the discriminative ability of the short FES-I and of each physical performance test between the two groups. The cut-off values for discrimination between the two groups of the short FES-I and each performance test were estimated using Youden’s index [40].

Furthermore, the associations between the short FES-I at baseline and the changes in each physical performance test at 1-year were investigated using multivariate linear regression analyses. The changes in physical performance over 1-year were calculated by subtracting physical performance at baseline from physical performance at 1 year. Multivariate linear regression analysis was performed, adjusting for potential confounding factors, with changes in each physical performance test at 1-year set as the dependent variable and the short FES-I set as the independent variable. On multivariate linear regression analysis, age, sex, physical performance at baseline, BMI, depressive symptoms, and pain were set as the confounding factors. All statistical analyses were performed using the R programming language and environment (R version 3.2.2) [41], with the level of significance at 5%.

## Results

The mean age ± standard deviation (SD) of the participants (75.9% women) in the present study was 71.1 ± 4.6 years, and 92.8% could perform IADL independently. In the comparison between follow-up subjects and non-follow-up subjects, no significant differences were found for all variables investigated in this study.

A total of 237 participants completed the 1-year follow-up survey and were included in the statistical analysis. The total number of falls during the 1-year follow-up period was 70, with a mean ± SD of 0.29 ± 0.80 times per person per year. Overall, 17.7% (42 persons) had at least one or more falls during the year, and 6.3% (15 persons) were recurrent fallers. Actual numbers of injurious falls were not available in this study. The descriptive statistics for the short FES-I, each physical performance test, and the confounding factors are presented in Table 1.

**Table 1:** Participants’ characteristics and factors associated with the number of falls during 1-year follow-up

		Overall	Number of falls in 1 y		
		*n* = 237	RR	95%CI	*p* value
Age (years)	mean ± SD	71.4 ± 4.6	NA	NA	NA
Sex (female)	n (%)	180(75.9%)	NA	NA	NA
Body mass index (kg/m^2^)	mean ± SD	22.3 ± 3.1	1.11	1.05: 1.18	<0.001
IADL (full marks)	n (%)	220 (92.8%)	0.57	0.27: 1.21	0.142
Depressive symptom (≥2 points)	n (%)	40(16.9%)	1.93	1.14: 3.28	0.015
Fall history (≥1 falls)	n (%)	21(8.9%)	2.91	1.64: 5.16	<0.001
Falls-related efficacy					
Short FES-I (points)	mean ± SD	11.7 ± 3.6	1.09	1.03: 1.15	0.001
Physical performance	mean ± SD				
5CWT (sec)	mean ± SD	3.4 ± 0.5	1.92	1.22: 3.03	0.005
5MWT (sec)	mean ± SD	2.6 ± 0.4	1.44	0.82: 2.54	0.207
FTSTS (sec)	mean ± SD	6.4 ± 1.9	1.07	0.97: 1.17	0.193
Grip strength (kg)	mean ± SD	26.8 ± 6.4	0.94	0.89: 1.00	0.060
TUGT (sec)	mean ± SD	5.7 ± 0.9	1.25	1.00: 1.57	0.053
Pain					
Knee pain	n (%)	90(38.0%)	2.39	1.48: 3.86	<0.001
Low back pain	n (%)	84(35.4%)	1.72	1.07: 2.77	0.025
Medication and medical history					
Number of medications (types/day)	mean ± SD	1.2 ± 1.0	1.32	1.06: 1.66	0.015
Diabetes mellitus	n (%)	22(9.3%)	1.08	0.49: 2.40	0.844
Heart disease	n (%)	20(8.4%)	2.03	1.03: 4.02	0.042
Respiratory disease	n (%)	17(7.2%)	2.41	1.22: 4.76	0.011
Stroke	n (%)	7(3.0%)	3.22	1.39: 7.45	0.006

*NA* Not applicable, *RR* relative risk adjusted for age and sex, 95%CI:95% confidence interval, *IADL* Instrumental Activities Of Daily Living, *Short FES-I* Short Falls Efficacy Scale International, *5CWT* 5-m Comfortable Pace Walking Time, *5MWT* 5-m Maximum Pace Walking Time, *FTSTS* 5 Times Sit To Stand Test, *TUGT* Timed Up And Go Test (sec)

**Fig. 1:**
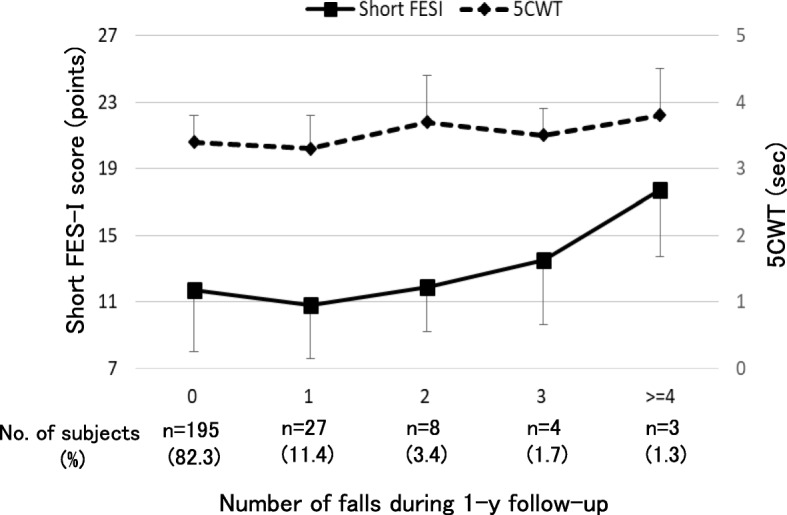
Associations of the short FES-I and 5CWT with the number of falls. The bold line and the dashed line represent the score of the short FES-I and the 5-m Comfortable Pace Walking Time (5CWT), respectively. On Poisson regression analysis adjusted for all potential confounding factors, the short FES-I score increases significantly according to the increase in the number of falls (RR = 1.09, *p* = 0.015). On the other hand, 5CWT is not significantly associated with the number of falls (RR = 1.55, *p* = 0.055)

With respect to the associations between the short FES-I score, each performance test, and the number of falls, the short FES-I score was significantly associated with the number of falls during the year on Poisson regression analysis adjusted for age and sex (relative risk (RR) =1.09, *p* < 0.001). Similarly, 5CWT was also significantly associated with the number of falls (RR = 1.92, *p* < 0.01). That is, a higher short FES-I score and a longer 5CWT each increased the number of falls (Fig. 1). On the other hand, grip strength and TUGT tended to be associated with the number of falls, but not significantly (*p* < 0.1). Including the above results, the factors associated with the number of falls during the 1-year follow-up are presented in Table 1. In summary, in addition to the short FES-I score and 5CWT, BMI, depressive symptoms, fall history, pain, medication, and medical history were found to be associated with the number of falls. Thus, these factors were all included as potential confounding factors in the Poisson regression analysis model. On Poisson regression analysis adjusted for age, sex, and all potential confounding factors, independent of all physical performance tests, the short FES-I was still found to be significantly associated with the number of falls during the 1-year follow-up (RRs were from 1.08 to1.09, *p* < 0.05) (Table 2). On the other hand, all physical performance tests were not significantly associated with the number of falls. Furthermore, the short FES-I score was found to discriminate significantly between recurrent fallers and non-recurrent fallers based on the ROC curve analysis (AUC = 0.65, *p* = 0.03); however, the difference between the two groups could not be significantly discriminated by all physical performance tests (Table 3). The cut-off value of the short FES-I for the discrimination of recurrent fallers was estimated to be 13 points (sensitivity = 0.60, specificity = 0.64). Even when the short FES-I score was transformed to a dichotomous variable according to a cut-off point of 13 points (low/high efficacy groups), the categorized short FES-I was still significantly associated with the number of falls (adjusted RRs ranged from 1.87 to 1.93, *p* < 0.05).

**Table 2:** Associations among the short FES-I, physical performance tests, and number of falls

Independent variable	Number of falls in 1 y		
	RR	95%CI	p value
Short FES-I and 5CWT			
Short FES-I (points)	1.09	1.02: 1.16	0.015
5CWT (sec)	1.55	0.99: 2.41	0.055
Goodness of fit test	χ^2^ = 205.25 (df = 223), *p* = 0.797		
Short FES-I and 5MWT			
Short FES-I (points)	1.09	1.02: 1.16	0.016
5MWT (sec)	1.08	0.62: 1.87	0.790
Goodness of fit test	χ^2^ = 208.6 (df = 223), *p* = 0.746		
Short FES-I and FTSTS			
Short FES-I (points)	1.09	1.02: 1.16	0.015
FTSTS (sec)	0.99	0.88: 1.12	0.924
Goodness of fit test	χ^2^ = 208.7 (df = 223), p = 0.746		
Short FES-I and grip strength			
Short FES-I (points)	1.08	1.01: 1.16	0.023
Grip strength (kg)	0.97	0.91: 1.03	0.326
Goodness of fit test	χ^2^ = 207.8 (df = 223), *p* = 0.760		
Short FES-I and TUGT			
Short FES-I (points)	1.08	1.01: 1.16	0.018
TUGT (sec)	1.06	0.88: 1.35	0.655
Goodness of fit test	χ^2^ = 208.5 (df = 223), *p* = 0.748		

*RR* relative risk adjusted for age, sex, BMI, depressive symptoms, fall history, knee pain, low back pain, number of medications, respiratory disease, heart disease, and stroke

*95%CI* 95% confidence interval, *Short FES-I* Short Falls Efficacy Scale International, *5CWT* 5-m Comfortable Pace Walking Time, *5MWT* 5-m Maximum Pace Walking Time, *FTSTS* 5 Times Sit To Stand Test, *TUGT* Timed Up And Go Test

**Table 3:** Comparison of recurrent-fallers to non-recurrent fallers using ROC curves for the short FES-I and physical performance tests

	Sensitivity	Specificity	Cut off	AUC [95%CI]	p value
Short FES-I (points)	0.600	0.644	13.00	0.652 [0.515: 0.789]	0.030
5CWT (sec)	0.933	0.324	3.15	0.631 [0.483: 0.779]	0.082
5MWT (sec)	0.800	0.311	2.45	0.518 [0.365: 0.672]	0.814
FTSTS (sec)	0.467	0.775	7.20	0.569 [0.394: 0.743]	0.442
TUGT (sec)	0.267	0.968	7.29	0.594 [0.425: 0.764]	0.275
Grip strength (kg)	0.267	0.838	32.6	0.506 [0.341: 0.670]	0.951

*Short FES-I* short Falls Efficacy Scale International, *5CWT* 5-m Comfortable Pace Walking Time, *5MWT* 5-m Maximum Pace Walking Time, *FTSTS* 5 Times Sit To Stand Test, *TUGT* Timed Up And Go Test

**Fig. 2:**
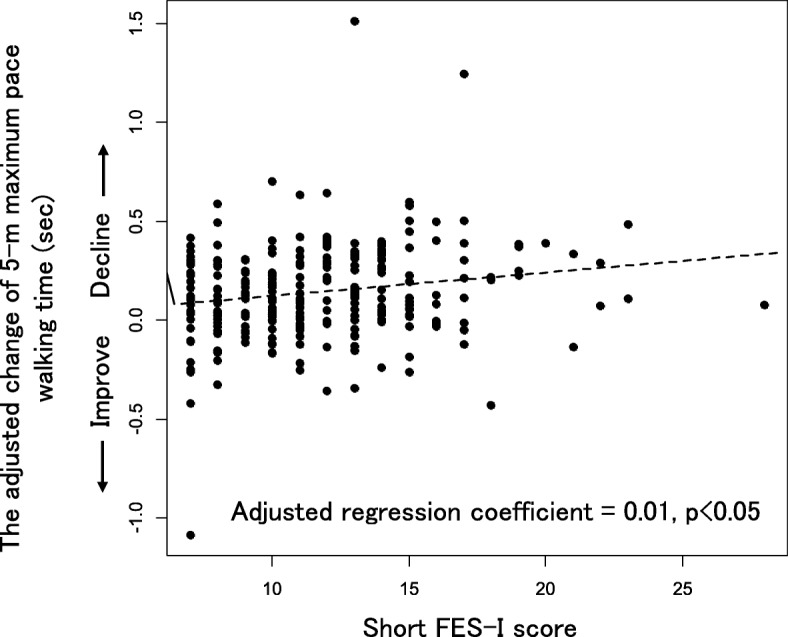
Scatter plot between the change in 5-m Maximum Pace Walking Time at 1-year and the short FES-I score. The short FES-I is significantly associated with the change in 5-m Maximum Pace Walking Time (5MWT) at 1-year on univariate and multivariate linear regression analyses. Age, sex, 5MWT at baseline, BMI, depressive symptoms, knee pain, and low back pain were set as the confounding factors in the multivariate linear regression analysis

As for the associations between the short FES-I at baseline and the changes in each physical performance test at 1-year, the short FES-I at baseline was significantly associated with the change in 5MWT at 1-year (adjusted regression coefficient = 0.01, *p* = 0.006), even with adjustment for potential confounding factors on multivariate linear regression analysis. That is, the higher the short FES-I score was, the greater was the decline in 5MWT at 1 year (Fig. 2).

## Discussion

The present longitudinal observational study examined whether fall-related efficacy was, independent of physical performance and other potential risk factors, associated with the occurrence of falls in community-dwelling older people.

In this study, about half of the participants could not be followed at the 1-year follow-up survey. However, no differences between follow-up subjects and non-follow-up subjects were found on statistical analysis; thus, any bias between the groups appeared to be negligible and could be ignored. Furthermore, as for the characteristics of the participants, almost all of the participants (about 93%) could perform IADL independently, and their mean 5CWT and TUGT results were faster than the reference values for Japanese older people [42, 43]. Therefore, the participants of the present study included a large number of older individuals with high functional capacity.

This longitudinal study found that the short FES-I score was significantly associated with future falls in community-dwelling older people, even with adjustment for the effects of both physical performance and other potential risk factors. Furthermore, it was shown that the short FES-I could discriminate better between recurrent fallers and non-recurrent fallers than physical performance. Thus, low fall-related efficacy was found to be associated with the occurrence of falls in community-dwelling older people, independent of physical performance and other potential risk factors. As in the present study, several prospective studies of community-dwelling older people also showed the association between fall-related efficacy and the occurrence of future falls [17–20]. On the other hand, various factors such as physical performance, depression, ADL, history of falls, and polypharmacy were identified as risk factors for falls in previous studies [29]; thus, the association between fall-related efficacy and the occurrence of future falls could have been affected by the interaction of physical performance and other potential risk factors. However, the effect of interaction among risk factors on the occurrence of falls was not considered in these previous studies. Consequently, the finding of the present study clarifies that assessment of fall-related efficacy is, independent of physical performance and other risk factors, a useful index of fall risk in community-dwelling older people.

Even though physical performance is a well-known risk factor for falling [29, 44, 45], physical performance was not associated with the number of falls in the present longitudinal study. The previous studies that investigated both fall-related efficacy and physical performance reported that both self-efficacy and physical performance were associated with the occurrence of falls [20, 46]. However, the participants of these previous studies were assumed to be relatively frail in comparison to the participants of the present study, and the effects of potential risk factors on the association of fall-related efficacy and falls were also not sufficiently considered. As described above, the participants of the present study were older people who could perform IADL independently and had a high level of physical performance. A systematic review reported previously indicated that the usefulness of the TUGT for prediction of falls was extremely limited for community-dwelling older people [47]. That is, in older people with high functional capacity, it was suggested that the association between physical performance and falling was difficult to demonstrate. In addition, physical function that could not be detected by measurement of physical performance tests might reflect on fall-related efficacy. For example, a meta-analysis indicated that fall-related efficacy is significantly associated with gait variability, which is a relevant marker of gait stability and cortical gait control [48]. Furthermore, older people with low fall-related efficacy even with high physiological function appear to have a fall risk from the neuropsychological perspective [20]. Therefore, the association between fall-related efficacy and the occurrence of falls appears to be a feature of community-dwelling older people with high functional capacity, and assessment of fall-related efficacy is a useful index to complement physical performance tests and assessments of other risk factors.

Furthermore, the short FES-I at baseline was shown to be associated with the change in 5MWT at 1-year in the present study. The relationships between fall-related efficacy and physical performance have been reported by many cross-sectional studies [7, 12, 25, 26, 31]. In addition, a previous study in which older people who visited an emergency department due to a fall-related injury were included reported that fall-related efficacy was related to future walking speed [27]. The findings of the present longitudinal observational study also showed that fall-related efficacy was related to the decrease of physical performance at 1-year in community-dwelling older people. Therefore, in community-dwelling older people with a high functional capacity, fall-related efficacy seems to be a useful index for predicting future decline of physical performance.

The present study had several limitations. First, the participants included only Japanese older people. The association between fall-related efficacy and falls has been shown to differ among cultures [14, 19]. Therefore, one cannot with confidence generalize the findings of the present study to older people in other countries. Second, fall-related efficacy was assessed by the short FES-I in the present study. However, in addition to the short FES-I, several scales that assess fall-related efficacy in older people have been developed and validated [7–11]. The short FES-I and the FES-I are suggested as appropriate scales for older people with high functional capacity [31]. If the other scales for fall-related efficacy were to be used, no association between efficacy and falls might be found. Third, the present study’s participants were older people with high functional capacity. If frail older people were to participate, one cannot know whether the association between efficacy and falls would remain. Fourth, the follow-up rate of the participants was low. As mentioned above, bias due to the low follow-up rate could be ignorable, but one cannot say that the low follow-up rate had completely no effect on the results of this study. Fifth, the data for injurious falls could not be obtained in this study. In particular, falls causing serious injuries, such as fractures, possibly lead to functional decline in older people. Therefore, to clarify the predictive ability of the short FES-I for injurious falls remains an issue, and further study is needed to address this point. Finally, based on the AUC of the ROC curve, the discrimination ability for recurrent fallers of the short FES-I was not acceptable [49]. Therefore, to enhance predictive ability for fall risk, establishment of a predictive model that considers other risk factors is necessary. Further study is needed to address this point.

## Conclusions

The present study showed that the short FES-I, independent of physical performance, is a useful index to detect fall risk in Japanese older people, and that fall-related efficacy is an important factor in terms of fall prevention.

## Data Availability

The datasets generated and analyzed during the current study are available from the corresponding author on reasonable request.

## Abbreviations

5CWT: 5-m Comfortable Pace Walking Time
5-GDS: five-item version of the Geriatric Depression Scale
5MWT: 5-m Maximum Pace Walking Time
ADL: Activities of daily living
BMI: Body mass index
FES-I: Falls Efficacy Scale International
FTSTS: 5 Times Sit to Stand Test
TUGT: Timed Up and Go Test
